# An interpretable machine-learning model for post-admission reassessment of bloodstream infection risk in ICU patients with pneumonia

**DOI:** 10.1038/s41598-026-54227-3

**Published:** 2026-06-06

**Authors:** Binglin Song, Xiangmei Yang, Wen Jin Wang, Kangrui Fu, Ping Liu, Chun Liu

**Affiliations:** 1 Clinical Medical College of North Sichuan Medical College, Nanchong, 637000 People’s Republic of China; 2 Emergency Department of Dazhou Central Hospital, No. 156, Dongda Road, Tongchuan District, Dazhou, 635000 Sichuan Province People’s Republic of China

**Keywords:** Intensive Care Unit (ICU), Pneumonia, Bloodstream Infection (BSI), Shapley Additive Explanations (SHAP), Machine Learning, Medical research, Risk factors

## Abstract

The aim of this study is to develop and validate a machine learning-based predictive model to assess the risk of acquired bloodstream infection (BSI) in ICU pneumonia patients. Data were obtained from the MIMIC-IV database and Dazhou Central Hospital. The MIMIC-IV cohort was randomly divided into a training set and an internal test set, and the Dazhou cohort served as an external validation set. Candidate predictors included demographic variables, comorbidities, severity scores, microbiological results, and ICU-course variables. Boruta feature selection followed by univariable and multivariable logistic regression was used to identify predictors for model development. To address class imbalance, SMOTE was applied during model training, and model discrimination was evaluated by the area under the receiver operating characteristic curve (AUC). SHAP was used to interpret the final model. Because several leading predictors in the primary model were only available after ICU admission, we additionally performed an admission-only sensitivity analysis after excluding post-admission variables such as ICU length of stay and sputum culture results. The final post-admission model incorporated six predictors: ICU length of stay, sputum culture for Gram-negative bacteria, sputum culture for Gram-positive cocci, APSIII score, SOFA score, and liver disease. Among the evaluated algorithms, the SMOTE-GBM model showed the best discrimination, with an AUC of 0.753 (95% CI 0.719–0.788) in the internal test set and 0.703 (95% CI 0.576–0.830) in the external validation set. SHAP analysis showed that ICU length of stay and sputum culture results were the major contributors to the post-admission model predictions. In the admission-only sensitivity analysis, model discrimination decreased, with the best AUC reaching 0.751 (95% CI 0.716–0.785) in the internal test set and 0.632 (95% CI 0.493–0.770) in the external validation set, indicating that early prediction using admission-available variables alone remained challenging. An interpretable post-admission machine-learning model showed moderate discrimination for reassessing BSI risk in ICU patients with pneumonia; however, limited external precision and the weaker performance of the admission-only sensitivity analysis indicate that further refinement is required before clinical implementation, especially for early decision-making.

## Objective

Every year, approximately 15 million patients are hospitalized due to pneumonia, with the reported incidence of bloodstream infections (BSI) varying based on the severity of the patients’ conditions. Intensive Care Unit (ICU) pneumonia patients are at a high risk of BSI due to their critical condition, compromised immune status, and the frequent need for invasive procedures^1^. Studies have reported that the incidence of BSI in ICU settings can range from 5% to 15%^2,3^, with the crude mortality rate for ICU patients with BSI exceeding 30%^4–6^. To effectively use antibiotics and monitor resistance, relevant research suggests that blood cultures should be performed on all hospitalized pneumonia patients^7,8^. This practice helps clinicians choose appropriate treatment regimens and provides more precise data on antimicrobial resistance.

Blood culture remains the gold standard for diagnosing BSI, but it typically takes 1 to 5 days to identify microorganisms, with a positive rate of only 10% to 20%^9,10^. The waiting time and low positivity rate of conventional blood cultures limit the timely escalation or de-escalation of antibiotics, contributing to the development of resistance and adverse outcomes^11^. Chalasani et al. found that 4.8% of blood culture samples from pneumonia patients were contaminated, a rate similar to that of pathogen identification^12^. Contaminated blood samples not only increase healthcare costs but also prolong patient hospitalization^13,14^. Moreover, researchers have raised questions about the necessity of performing blood cultures on all hospitalized pneumonia patients^15,16^. Waterer and Wunderink pointed out that, according to Fine’s Pneumonia Severity Index (PSI)^17^, the incidence of BSI is significantly lower in low-risk patients compared to those with severe conditions^18–20^. Therefore, improved risk stratification for BSI may help support microbiological evaluation and antimicrobial stewardship; however, the usefulness of any prediction model depends on whether its predictors are available at the time the clinical decision is being made.

Machine learning (ML) is a computer algorithm that learns from data and identifies potential patterns, enabling the discovery of complex relationships within large datasets, which has significant clinical value^21,22^. However, ML models are often referred to as “black boxes,” as the relationship between input and output cannot be easily explained, making them difficult to apply in clinical practice^23^. Shapley additive explanations (SHAP), as a post hoc explanation algorithm, can map SHAP values to ML models using additive attribution, allowing effective interpretation and visualization of ML models^24^.

Currently, no externally validated model has been reported specifically for estimating BSI risk in ICU patients with pneumonia. In this study, we developed and externally validated an interpretable machine-learning model using variables available during the early ICU course, including several post-admission predictors, to support post-admission risk reassessment rather than the initial decision to obtain blood cultures. In addition, to address concerns regarding predictor timing, we performed an admission-only sensitivity analysis restricted to variables available at or before ICU admission.

## Methods

### Data sources

This study is a retrospective analysis based on the MIMIC-IV database and data from a tertiary hospital in China. The MIMIC-IV database contains clinical information from all patients admitted to the Beth Israel Deaconess Medical Center between 2008 and 2022. This database includes a wealth of patient information, including hospital course, laboratory test results, medication usage, and vital signs^25^. To protect patient privacy, all personally identifiable information has been de-identified, and real identifiers are replaced with random codes. The researchers have completed the required training by the collaborating institution and have obtained permission for data usage and extraction (certificate number: 65828043).

Dazhou Central Hospital, located in Sichuan, China, is a tertiary hospital. The validation data comes from the ICU of Dazhou Central Hospital, which has 70 beds. Retrospective data from patients admitted to the ICU between January 1, 2019, and May 1, 2025, were collected, and informed consent was obtained from the hospital’s Ethics Committee. To ensure patient privacy, all personally identifiable information has been de-identified, and real identifiers are replaced with random codes. Therefore, informed consent from patients was not required for this study.

### Study population

Patients diagnosed with pneumonia who were admitted to the ICU were included. The pneumonia cohort from the MIMIC-IV database was initially identified using the International Classification of Diseases (ICD) codes recorded during their hospital stay. Specifically, we included patients with ICD-9-CM codes ranging from 480.0 to 486 and ICD-10-CM codes ranging from J12.0 to J18.9.Representative ICD codes used for inclusion include, but are not limited to: ICD-9-CM codes 482.4 (Pneumonia due to Streptococcus pneumoniae), 484.5 (Pneumonia in cytomegaloviral disease), and 486 (Pneumonia, organism unspecified); and ICD-10-CM codes J18.9 (Pneumonia, unspecified organism), J15.9 (Unspecified bacterial pneumonia), and J12.9 (Unspecified viral pneumonia).

Furthermore, to ensure clinical relevance and consistency with established guidelines, the final inclusion required the presence of new pulmonary infiltrates on chest X-ray or CT scan, along with at least one of the following acute lower respiratory tract infection symptoms: fever, sputum production, purulent sputum, dyspnea, pleuritic chest pain, focal chest signs, or abnormal peripheral white blood cell count^26^.

BSI (bloodstream infection) was defined as the isolation of a clinically significant pathogen from at least one blood culture bottle. According to the CDC/NHSN laboratory-confirmed bloodstream infection (LCBI) guidelines, potential contaminants (including coagulase-negative staphylococci, Bacillus species, Corynebacterium species, Propionibacterium species, and Cutibacterium species) are not considered BSI^27^.

Exclusion criteria included:①.Patients under 18 years of age;②.Patients with ICU stay < 24 h;③.Patients with multiple ICU admissions or positive blood cultures prior to ICU admission;④.Patients who did not undergo blood culture.

### Data collection

The following data were extracted from the MIMIC-IV database: ①.Demographic characteristics, including sex, age at admission, weight and height; ②.Vital signs data, including heart rate, respiratory rate, systolic blood pressure, mean arterial pressure, oxygen saturation, and maximum temperature; ③.Comorbidities, including hypertension, heart failure, chronic lung disease, diabetes, renal disease, severe liver disease, HIV, and malignancy; ④.Laboratory parameters within the first 24 h of ICU admission, including coagulation function, C-reactive protein (CRP), renal function, complete blood count, blood gas analysis, etc.; ⑤.Sputum and blood culture results; ⑥.Mechanical ventilation and invasive catheter data.

All extracted variables were initially considered as candidate predictors. Because these variables were not available at the same clinical time point, we prespecified two groups for interpretation: admission-available variables, which could be obtained at or before ICU admission or within the first 24 h, and post-admission variables, which became available later during the ICU stay.

### Data preprocessing

All data processing was performed in R version 4.4.1. Missing value analysis was conducted, and variables with missing data exceeding 30% were excluded. For variables with a missing rate below 30%, multiple imputation by chained equations (MICE) was applied to complete the dataset and reduce bias introduced by missing values^28,29^.To prevent the impact of inaccurate data caused by entry errors in the database, the first and last 1% of continuous variables were removed.

Because bloodstream infection was relatively infrequent, class imbalance was addressed during model development using the Synthetic Minority Oversampling Technique (SMOTE). This procedure was used to improve minority-class learning during training; however, model transportability was evaluated using non-synthetic validation data, and the potential influence of oversampling on generalizability was considered in the interpretation of results^30^.

### Statistical methods

Categorical data were expressed as counts and percentages. For continuous data with non-normal distribution, the median and interquartile range (IQR) were reported. Group differences were compared using analysis of variance (ANOVA) or the Mann-Whitney U test. All statistical tests were two-tailed, and a p-value of less than 0.05 was considered statistically significant.

### Clinical timing of predictor availability

For interpretation of the primary model, predictors were grouped according to clinical availability. Admission-available variables included baseline comorbidities and severity indicators assessed at or near ICU admission, such as liver disease, SOFA score, and APSIII score. Post-admission variables included ICU length of stay and microbiological results that became available later during hospitalization, such as sputum culture findings. Accordingly, the primary model should be interpreted as a post-admission reassessment model rather than an admission-time triage tool. To examine how this timing dependence affected performance, we additionally performed an admission-only sensitivity analysis after excluding post-admission predictors.

### Admission-only sensitivity analysis

To assess the dependence of model performance on post-admission information, we conducted an admission-only sensitivity analysis restricted to predictors available at or before ICU admission. Variables that became available later during the ICU course, including ICU length of stay and sputum culture results, were excluded. The restricted predictor set was then re-evaluated using the same model-development framework as in the primary analysis.

### Machine learning model construction and evaluation

The dataset was randomly divided into a training set (60%, *n* = 2473), a validation set (40%, *n* = 1648), and an external validation set (*n* = 150). First, the Boruta algorithm was used for feature selection to identify potential influencing factors. Then, univariate and multivariate logistic regression analyses were conducted to select statistically significant variables, ultimately determining the independent risk factors for BSI in pneumonia patients.

Eight machine learning models were used: Logistic Regression (LR), Gradient Boosting Machine (GBM), Neural Network (NN), Extreme Gradient Boosting (XGBoost), Adaptive Boosting (AdaBoost), Light Gradient Boosting Machine (LightGBM), Category Boosting (CatBoost), and Support Vector Machine (SVM). During model training, 10-fold cross-validation was used to enhance the model’s generalizability, and an automated hyperparameter optimization process was applied to fine-tune the models^31^.

The predictive performance of the models was evaluated on the validation set using metrics such as AUC-ROC, accuracy, precision, recall, and F1-score. Finally, the best-performing model was selected, and the importance of each feature in the black-box model was explained by calculating the SHAP (Shapley Additive Explanations) values.External validation was performed using data from Dazhou Central Hospital to examine generalizability and applicability in an independent cohort.

### SHAP interpretability analysis

To improve the interpretability of the model, we employed the SHAP method. SHAP, based on the concept of Shapley values from game theory, uses an additive model to assess the contribution of each feature to the model’s prediction, providing a deep understanding of the model’s output, especially for complex machine learning models^32^. We used the shapviz package to compute and visualize the SHAP values for the GBM model, thereby revealing the key factors associated with BSI in pneumonia patients.

This study interpreted and visualized the GBM model according to SHAP guidelines (https://github.com/slundberg/shap), generating SHAP histograms, SHAP beeswarm plots, and waterfall plots. A higher SHAP value indicates that the feature has a greater impact on the prediction. To illustrate, we visualized the GBM model based on the SHAP histogram and SHAP waterfall plots for one patient who developed BSI and one who did not.

## Results

### Baseline characteristics

A total of 8,971 pneumonia patients were extracted from the MIMIC database. After excluding 1,791 patients who did not undergo blood culture, 7,180 patients remained. After data preprocessing (removal of the first and last 1% of continuous variables), 4,121 patients from the MIMIC database met the inclusion criteria, including 468 BSI patients. The baseline characteristics are shown in Table 1.

**Table 1:** Baseline table.

Patients characteristics	MIMIC-IV cohort No Bloodstream Infections (*n* = 3653)	Bloodstream Infections (*n* = 468)	*P*	Hospital cohort Bloodstream Infections (*n* = 132)	Bloodstream Infections (*n* = 18)	*P*
Demographic data						
Gender, n (%)			0.23			0.416
Female	1468 (40)	174 (37)		54 (41)	5 (28)	
Male	2185 (60)	294 (63)		78 (59)	13 (72)	
Age, Median (Q1, Q3)	69.83 (58.64, 80.31)	67.3 (55.94, 75.12)	< 0.001	70.38 (58.75, 83.03)	67.68 (59.64, 77.49)	0.688
Weight, Median (Q1, Q3)	80 (66.95, 96)	85.1 (69.54, 102.6)	< 0.001	78.63 (62.75, 91.63)	87.65 (74.12, 99.55)	0.067
Score, Median (Q1, Q3)						
Sofa, Median (Q1, Q3)	5 (3, 8)	6 (4, 9)	< 0.001	5 (3, 7)	6 (2.25, 8)	0.843
apsiii, Median (Q1,Q3)	47 (36, 61)	54 (43.75, 70)	< 0.001	48 (37, 61)	52 (44.25, 66)	0.121
sapsii, Median (Q1, Q3)	39 (31, 48)	42 (33, 51)	< 0.001	39 (32, 47.25)	34 (28.5, 48.5)	0.459
Comorbidity, n (%)						
Hypertension, n (%)			0.999			0.165
No	1269 (35)	162 (35)		48 (36)	3 (17)	
Yes	2384 (65)	306 (65)		84 (64)	15 (83)	
Chronic rel disease, n (%)			0.624			0.54
No	2879 (79)	374 (80)		105 (80)	13 (72)	
Yes	774 (21)	94 (20)		27 (20)	5 (28)	
Cardiac failure, n (%)			0.678			1
No	3339 (91)	431 (92)		118 (89)	17 (94)	
Yes	314 (9)	37 (8)		14 (11)	1 (6)	
Chronic respiratory disease, n (%)			0.008			0.94
No	2501 (68)	349 (75)		90 (68)	13 (72)	
Yes	1152 (32)	119 (25)		42 (32)	5 (28)	
Diabetes, n (%)			0.007			0.992
No	2568 (70)	300 (64)		85 (64)	11 (61)	
Yes	1085 (30)	168 (36)		47 (36)	7 (39)	
Liver disease, n (%)			< 0.001			0.036
No	3166 (87)	377 (81)		115 (87)	12 (67)	
Yes	487 (13)	91 (19)		17 (13)	6 (33)	
HIV, n (%)			0.766			1
No	3626 (99)	466 (100)		130 (98)	18 (100)	
Yes	27 (1)	2 (0)		2 (2)	0 (0)	
CA, n (%)			0.016			0.202
No	2966 (81)	402 (86)		107 (81)	17 (94)	
Yes	687 (19)	66 (14)		25 (19)	1 (6)	
Vas agent, n (%)			< 0.001			1
No	2098 (57)	172 (37)		74 (56)	10 (56)	
Yes	1555 (43)	296 (63)		58 (44)	8 (44)	
Ventilation, n (%)			< 0.001			0.436
No	1537 (42)	100 (21)		61 (46)	6 (33)	
Yes	2116 (58)	368 (79)		71 (54)	12 (67)	
Invasive line, n (%)			< 0.001			1
No	1309 (36)	93 (20)		45 (34)	6 (33)	
Yes	2344 (64)	375 (80)		87 (66)	12 (67)	
Sputum culture results were fungal, n (%)			< 0.001			1
No	2986 (82)	296 (63)		103 (78)	14 (78)	
Yes	667 (18)	172 (37)		29 (22)	4 (22)	
Sputum cultures revealed Gram-positive cocci, n (%)			< 0.001			0.185
No	3235 (89)	351 (75)		112 (85)	13 (72)	
Yes	418 (11)	117 (25)		20 (15)	5 (28)	
Sputum cultures showed gram-negative bacilli, n (%)			< 0.001			0.482
No	3076 (84)	302 (65)		113 (86)	14 (78)	
Yes	577 (16)	166 (35)		19 (14)	4 (22)	
aki, n (%)			< 0.001			0.123
No	533 (15)	29 (6)		30 (23)	1 (6)	
Yes	3120 (85)	439 (94)		102 (77)	17 (94)	
Los icu, day (Q1,Q3)	4.58 (2.34, 9.45)	10.3 (4.46, 20.35)	< 0.001	4.39 (2.19, 9.39)	4.12 (2.95, 15.23)	0.289
Vital signs, Median (Q1, Q3)						
Resp rate, breaths/min	20.85 (18.27, 23.81)	21.2 (18.6, 24.28)	0.146	21.41 (18.91, 23.61)	21.78 (20.06, 26.2)	0.489
Temperature, ℃	36.93 (36.69, 37.29)	37.06 (36.73, 37.42)	< 0.001	36.97 ± 0.41	37.01 ± 0.52	0.726
spo2, %	96.19 (94.83, 97.55)	96.51 (95.17, 97.77)	0.003	96.16 ± 1.69	96.53 ± 1.69	0.403
Heart rate, beats/min	86.92 (77.07, 98.58)	89.42 (78.44, 100.23)	0.03	89.78 ± 14.22	82.56 ± 16.38	0.09
sbp, mmHg	115.26 (106.57, 126.46)	113.48 (106.13, 123.93)	0.137	117.16 (107.77, 130.03)	117.49 (103.48, 132.13)	0.688
dbp, mmHg	61.73 (55.94, 68.75)	60.89 (55.36, 67.8)	0.209	63.38 (56.47, 70.85)	61.11 (53.89, 71.3)	0.461
Laboratory results, Median (Q1, Q3)						
inr,	1.3 (1.2, 1.6)	1.3 (1.2, 1.6)	0.528	1.3 (1.2, 1.6)	1.35 (1.2, 1.6)	0.499
pt, sec	14.4 (12.8, 17.3)	14.55 (12.88, 17.6)	0.29	14.2 (12.78, 17.65)	14.85 (13.12, 17.27)	0.658
ptt, sec	31.3 (27.5, 38.2)	31.9 (27.9, 42.3)	0.036	31 (27, 37.12)	32.8 (26.67, 39.83)	0.532
ph,	7.38 (7.31, 7.43)	7.37 (7.3, 7.42)	0.048	7.38 (7.32, 7.43)	7.4 (7.34, 7.44)	0.207
Aniongap, mmol/L	14 (12, 17)	15 (12, 17)	0.565	15 (12, 17)	14.5 (13, 17)	0.448
Bicarbote, mmol/L	23 (20, 26)	22 (19, 25)	< 0.001	22 (20, 25)	21 (20, 24.5)	0.437
Bun, mg/dL	24 (16, 38)	26 (17, 42.25)	0.003	21.5 (16, 40)	24.5 (17.75, 37.5)	0.696
Creatinine, mg/dL	1.1 (0.8, 1.6)	1.2 (0.9, 2)	0.001	1.1 (0.8, 1.52)	1.3 (0.72, 2.15)	0.472
Glucose, mg/dL	133 (109, 173)	137 (112, 186)	0.041	134 (108, 183.25)	143.5 (118, 189.75)	0.892
Bilirubin, µmol/L	0.6 (0.4, 1.1)	0.7 (0.4, 1.2)	0.02	0.6 (0.4, 1.2)	0.8 (0.52, 1.62)	0.234
wbc, 10⁹/L	11.7 (8.3, 16.3)	12 (8.4, 16.7)	0.387	11.8 (9.6, 16.62)	12.55 (6.97, 15.65)	0.515
Hematocrit, %	34.4 (29.2, 39.6)	34.8 (29.48, 39.6)	0.692	35.54 ± 7.39	36.11 ± 7.34	0.76
Platelet, 10⁹/L	208 (148, 282)	199 (133, 264.75)	0.003	226 (152, 289.25)	202 (157.25, 245.75)	0.479
Hemoglobin, g/dL	11.3 (9.4, 13)	11.25 (9.6, 12.9)	0.573	11.55 ± 2.43	11.84 ± 2.08	0.582
mch, pg	30.1 (28.6, 31.7)	30 (28.5, 31.7)	0.769	30.2 (29.08, 31.3)	31 (29.42, 31.8)	0.212
mchc, g/dL	32.7 (31.5, 33.7)	32.7 (31.7, 33.8)	0.476	32.52 ± 1.51	32.61 ± 1.71	0.823
mcv, fL	92 (88, 96)	92 (88, 96)	0.366	93 (89, 96)	93.5 (90.25, 101.5)	0.32
rbc, 10¹²/L	3.76 (3.16, 4.33)	3.8 (3.2, 4.32)	0.51	3.84 ± 0.85	3.85 ± 0.77	0.956
rdw, %	14.6 (13.6, 16.3)	14.6 (13.6, 16.3)	0.752	14.75 (13.8, 16.35)	14.35 (13.35, 18.2)	0.945
po2, mmHg	76 (50, 126)	80 (53, 155.25)	0.028	74 (46.75, 122.5)	86 (64.5, 197)	0.271
pco2, mmHg	42 (36, 50)	41 (36, 51)	0.417	44 (36, 51)	39.5 (34.5, 45.75)	0.203
Total co2, mmol/L	25 (22, 29)	24 (21, 28)	< 0.001	25 (22, 29)	23.5 (20, 26)	0.108

**Fig. 1:**
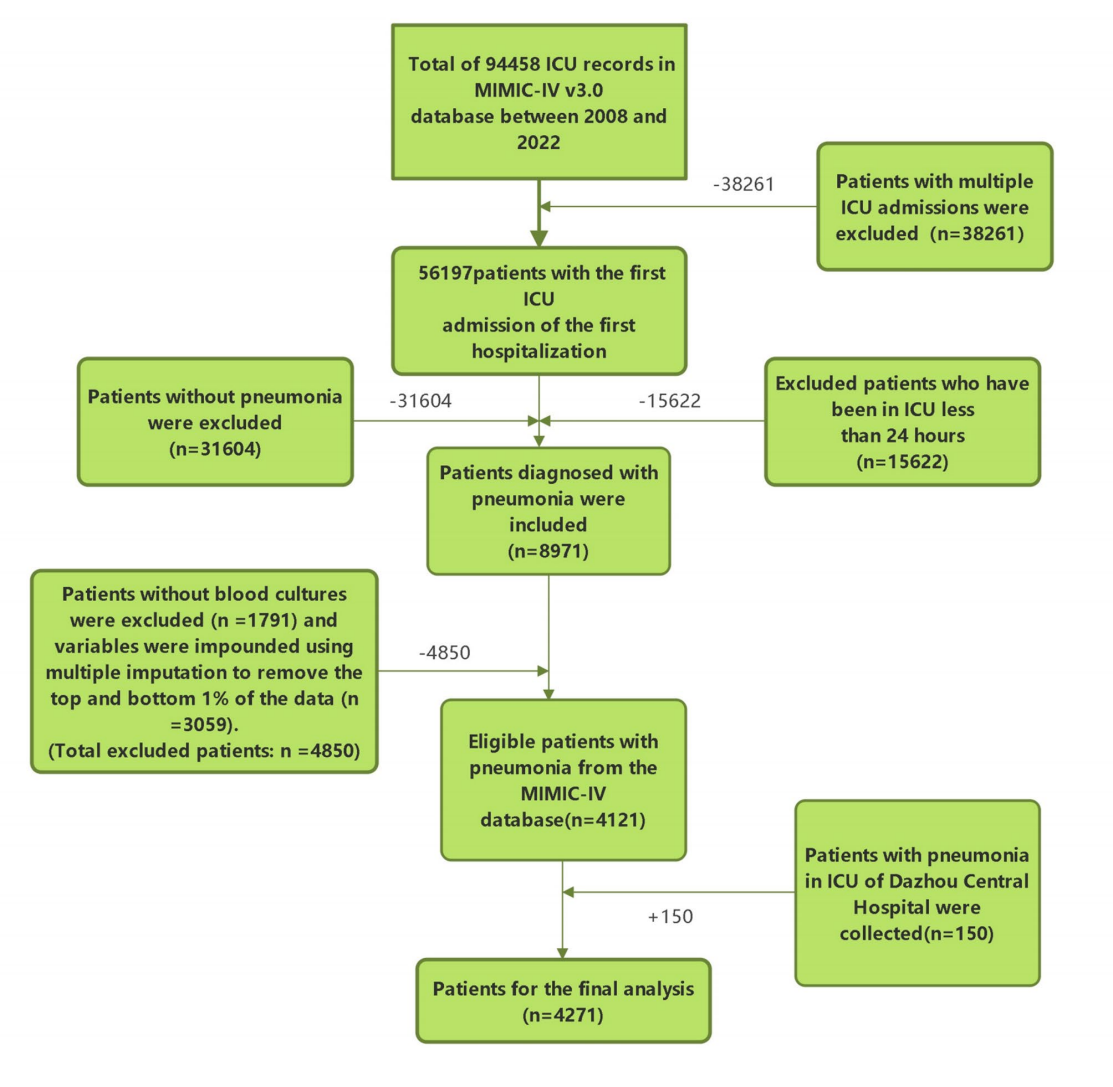
Flow chart showing the data preparation process (This figure depicts the patient selection flow from the MIMIC-IV database, focusing on pneumonia cases in ICU settings. Patients with multiple ICU admissions, those without pneumonia, stays under 24 h, or missing blood culture data were excluded. The final cohort consists of pneumonia patients, including data from Dazhou Central Hospital ICU.)

A total of 150 eligible patients were included from Dazhou Central Hospital, including 18 BSI patients. The patient inclusion flowchart is shown in Fig. 1.

### Model development and validation

**Fig. 2:**
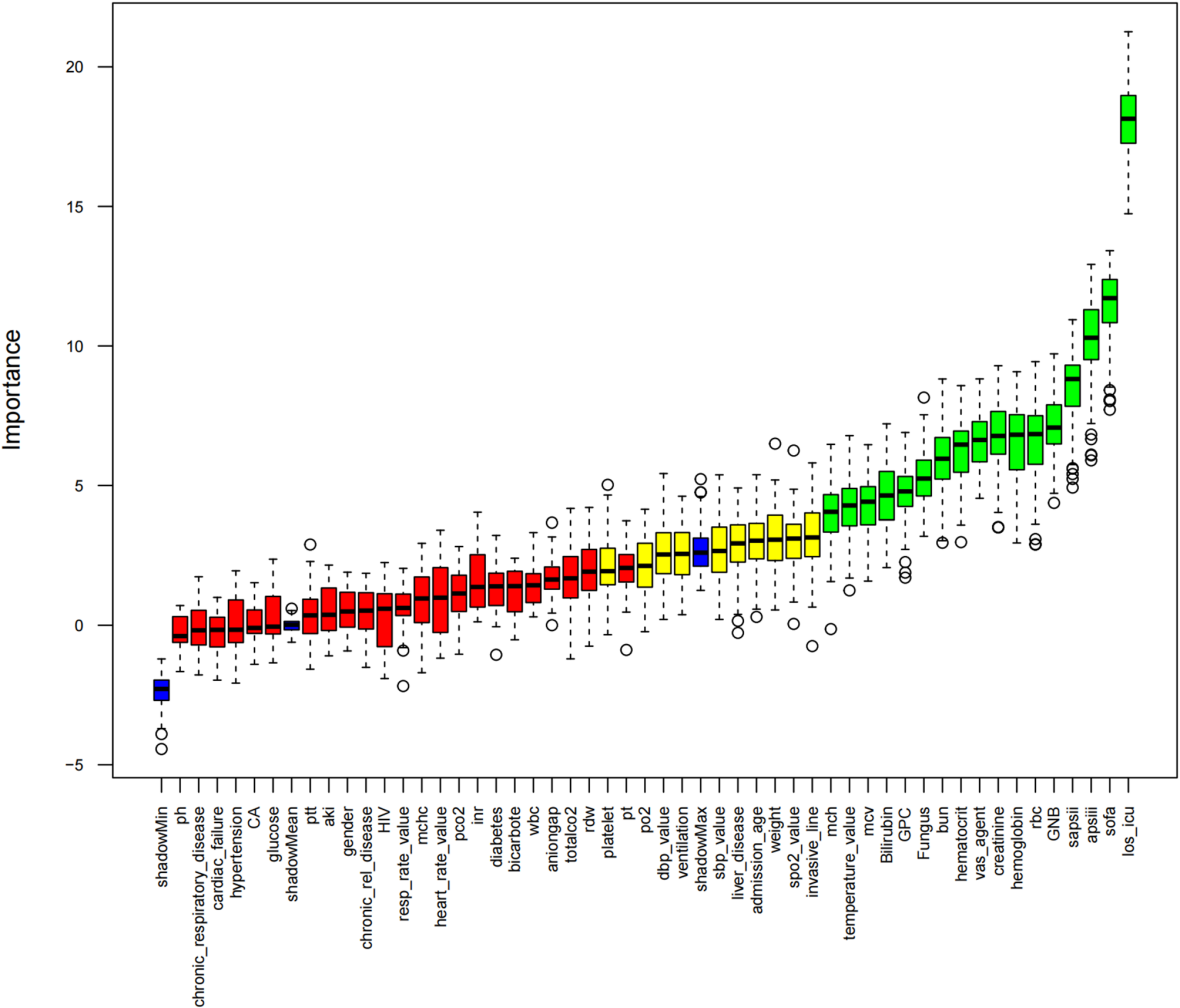
Variable selection for ICU pneumonia analysis using the Boruta method (This figure illustrates the process of variable selection for ICU pneumonia analysis using the Boruta method. The features are ranked by importance, with red indicating low importance and green indicating high importance. The boxplot displays the distribution of each feature’s importance value, highlighting the variables that have the greatest impact on the model’s predictions).

Through the Boruta algorithm and univariate and multivariate logistic regression, 6 important variables for identifying bloodstream infections (BSI) were selected from 50 clinical features. These variables include ICU length of stay, sputum culture for Gram-negative bacteria (Sputum-GNB), sputum culture for Gram-positive cocci (Sputum-GPC), APSIII score, SOFA score, and liver injury (Fig. 2; Tables 2 and 3).

**Table 2:** Variables with statistical significance in the results of univariate and multivariate logistic regression analysis. *p* < .05.

Name	desc	OR (univariable)	OR (multivariable)
los_icu	Mean ± SD	1.07 (1.05–1.08, *p*<.001)	1.05 (1.03–1.06, *p*<.001)
sofa	Mean ± SD	1.08 (1.04–1.11, *p*<.001)	0.93 (0.87–0.99, *p*=.021)
apsiii	Mean ± SD	1.02 (1.01–1.02, *p*<.001)	1.01 (1.00-1.02, *p*=.032)
liver_disease	No		
	Yes	1.51 (1.10–2.08, *p*=.012)	1.50 (1.02–2.21, *p*=.039)
Sputum-GPC	No		
	Yes	2.67 (1.98–3.59, *p*<.001)	1.68 (1.20–2.34, *p*=.002)
Sputum-GNB	No		
	Yes	2.78 (2.12–3.64, *p*<.001)	1.56 (1.14–2.13, *p*=.006)

**Table 3:** Variable screening based on univariate and multivariate logistic, *p* < .05 is statistically significant.

Name	desc	No Bloodstream Infections (*N* = 2192)	Bloodstream Infections (*N* = 281)	id	OR (univariable)	OR (multivariable)
Hypertension	No	757 (34.5%)	96 (34.2%)	hypertensionNo		
	Yes	1435 (65.5%)	185 (65.8%)	hypertensionYes	1.02 (0.78–1.32, *p*=.902)	
Chronic rel disease	No	1735 (79.2%)	223 (79.4%)	chronic rel diseaseNo		
	Yes	457 (20.8%)	58 (20.6%)	chronic rel diseaseYes	0.99 (0.73–1.34, *p*=.936)	
Cardiac failure	No	2007 (91.6%)	257 (91.5%)	cardiac failureNo		
	Yes	185 (8.4%)	24 (8.5%)	cardiac failureYes	1.01 (0.65–1.58, *p*=.954)	
Chronic respiratory disease	No	1493 (68.1%)	213 (75.8%)	chronic respiratory diseaseNo		
	Yes	699 (31.9%)	68 (24.2%)	chronic respiratory diseaseYes	0.68 (0.51–0.91, *p*=.009)	0.75 (0.55–1.03, *p*=.079)
Diabetes	No	1537 (70.1%)	185 (65.8%)	diabetesNo		
	Yes	655 (29.9%)	96 (34.2%)	diabetesYes	1.22 (0.94–1.58, *p*=.142)	
Liver disease	No	1894 (86.4%)	227 (80.8%)	liver diseaseNo		
	Yes	298 (13.6%)	54 (19.2%)	liver diseaseYes	1.51 (1.10–2.08, *p*=.012)	1.50 (1.02–2.21, *p*=.039)
HIV	No	2176 (99.3%)	280 (99.6%)	HIVNo		
	Yes	16 (0.7%)	1 (0.4%)	HIVYes	0.49 (0.06–3.68, *p*=.484)	
CA	No	1787 (81.5%)	247 (87.9%)	CANo		
	Yes	405 (18.5%)	34 (12.1%)	CAYes	0.61 (0.42–0.88, *p*=.009)	0.80 (0.53–1.21, *p*=.288)
Vas agent	No	1238 (56.5%)	103 (36.7%)	vas agentNo		
	Yes	954 (43.5%)	178 (63.3%)	vas agentYes	2.24 (1.73–2.90, *p*<.001)	1.36 (0.94–1.98, *p*=.100)
Ventilation	No	907 (41.4%)	58 (20.6%)	ventilationNo		
	Yes	1285 (58.6%)	223 (79.4%)	ventilationYes	2.71 (2.01–3.67, *p*<.001)	1.15 (0.77–1.71, *p*=.503)
Invasive line	No	793 (36.2%)	63 (22.4%)	invasive lineNo		
	Yes	1399 (63.8%)	218 (77.6%)	invasive lineYes	1.96 (1.46–2.63, *p*<.001)	1.08 (0.72–1.60, *p*=.711)
Sputum culture was fungal	No	1770 (80.7%)	179 (63.7%)	No		
	Yes	422 (19.3%)	102 (36.3%)	Yes	2.39 (1.83–3.12, *p*<.001)	1.22 (0.89–1.67, *p*=.207)
Sputum culture revealed Gram-negative bacilli	No	1937 (88.4%)	208 (74%)	No		
	Yes	255 (11.6%)	73 (26%)	Yes	2.67 (1.98–3.59, *p*<.001)	1.68 (1.20–2.34, *p*=.002)
Sputum culture revealed Gram-positive cocci	No	1838 (83.9%)	183 (65.1%)	No		
	Yes	354 (16.1%)	98 (34.9%)	Yes	2.78 (2.12–3.64, *p*<.001)	1.56 (1.14–2.13, *p*=.006)
aki	No	323 (14.7%)	21 (7.5%)	akiNo		
	Yes	1869 (85.3%)	260 (92.5%)	akiYes	2.14 (1.35–3.39, *p*=.001)	1.01 (0.61–1.67, *p*=.969)
Gender	Female	898 (41%)	101 (35.9%)	genderFemale		
	Male	1294 (59%)	180 (64.1%)	genderMale	1.24 (0.96–1.60, *p*=.107)	
Admission age	Mean ± SD	68.2 ± 15.4	66.1 ± 14.3	admission age	0.99 (0.98-1.00, *p*=.032)	1.00 (0.99–1.02, *p*=.450)
los icu	Mean ± SD	7.4 ± 7.8	15.7 ± 15.4	los icu	1.07 (1.05–1.08, *p*<.001)	1.05 (1.03–1.06, *p*<.001)
Weight	Mean ± SD	84.5 ± 25.0	89.3 ± 27.3	weight	1.01 (1.00-1.01, *p*=.003)	1.00 (1.00-1.01, *p*=.297)
inr	Mean ± SD	1.6 ± 0.9	1.6 ± 1.0	inr	1.00 (0.87–1.15, *p*=.995)	
pt	Mean ± SD	17.0 ± 9.0	17.5 ± 13.3	pt	1.01 (0.99–1.02, *p*=.343)	
ptt	Mean ± SD	37.9 ± 22.4	38.6 ± 20.2	ptt	1.00 (1.00-1.01, *p*=.622)	
ph	Mean ± SD	7.4 ± 0.1	7.4 ± 0.1	ph	0.21 (0.05–0.82, *p*=.024)	0.89 (0.18–4.44, *p*=.888)
aniongap	Mean ± SD	14.9 ± 3.8	15.0 ± 4.0	aniongap	1.01 (0.98–1.04, *p*=.518)	
bicarbote	Mean ± SD	23.2 ± 4.6	22.2 ± 4.4	bicarbote	0.95 (0.93–0.98, *p*=.002)	0.99 (0.94–1.04, *p*=.615)
Bun	Mean ± SD	30.4 ± 22.0	35.0 ± 27.0	bun	1.01 (1.00-1.01, *p*=.002)	1.01 (1.00-1.01, *p*=.131)
Creatinine	Mean ± SD	1.6 ± 1.5	1.8 ± 1.6	creatinine	1.08 (1.01–1.16, *p*=.027)	1.03 (0.92–1.16, *p*=.626)
Glucose	Mean ± SD	154.9 ± 86.9	157.7 ± 66.0	glucose	1.00 (1.00–1.00, *p*=.599)	
Bilirubin	Mean ± SD	1.4 ± 3.4	1.7 ± 4.0	Bilirubin	1.02 (0.99–1.05, *p*=.181)	
Wbc	Mean ± SD	13.5 ± 10.7	13.3 ± 8.4	wbc	1.00 (0.99–1.01, *p*=.788)	
Heart rate value	Mean ± SD	87.9 ± 14.9	90.2 ± 15.9	heart rate value	1.01 (1.00-1.02, *p*=.015)	1.01 (1.00-1.02, *p*=.051)
sbp value	Mean ± SD	117.5 ± 14.5	117.3 ± 14.8	sbp value	1.00 (0.99–1.01, *p*=.809)	
dbp value	Mean ± SD	62.8 ± 10.0	62.6 ± 10.0	dbp value	1.00 (0.99–1.01, *p*=.720)	
resp rate value	Mean ± SD	21.2 ± 4.0	21.6 ± 4.1	resp rate value	1.02 (0.99–1.05, *p*=.135)	
Temperature value	Mean ± SD	37.0 ± 0.5	37.1 ± 0.5	temperature value	1.53 (1.19–1.96, *p*<.001)	1.20 (0.90–1.60, *p*=.212)
spo2 value	Mean ± SD	96.2 ± 1.9	96.3 ± 1.9	spo2 value	1.03 (0.97–1.10, *p*=.326)	
Sofa	Mean ± SD	5.8 ± 3.5	6.8 ± 3.7	sofa	1.08 (1.04–1.11, *p*<.001)	0.93 (0.87–0.99, *p*=.021)
Apsiii	Mean ± SD	50.8 ± 19.7	58.0 ± 21.8	apsiii	1.02 (1.01–1.02, *p*<.001)	1.01 (1.00-1.02, *p*=.032)
Sapsii	Mean ± SD	40.4 ± 13.4	43.3 ± 13.5	sapsii	1.02 (1.01–1.02, *p*<.001)	1.00 (0.98–1.02, *p*=.979)
Hematocrit	Mean ± SD	34.5 ± 7.1	34.8 ± 6.9	hematocrit	1.00 (0.99–1.02, *p*=.578)	
Platelet	Mean ± SD	224.8 ± 115.7	209.8 ± 103.9	platelet	1.00 (1.00–1.00, *p*=.038)	1.00 (1.00–1.00, *p*=.135)
Hemoglobin	Mean ± SD	11.3 ± 2.4	11.4 ± 2.3	hemoglobin	1.02 (0.97–1.08, *p*=.450)	
Mch	Mean ± SD	30.1 ± 2.6	29.9 ± 2.8	mch	0.98 (0.93–1.02, *p*=.318)	
Mchc	Mean ± SD	32.6 ± 1.6	32.7 ± 1.7	mchc	1.02 (0.94–1.10, *p*=.619)	
Mcv	Mean ± SD	92.3 ± 7.0	91.6 ± 6.5	mcv	0.99 (0.97-1.00, *p*=.112)	
Rbc	Mean ± SD	3.8 ± 0.8	3.8 ± 0.8	rbc	1.10 (0.95–1.28, *p*=.204)	
Rdw	Mean ± SD	15.2 ± 2.3	15.2 ± 2.2	rdw	1.00 (0.95–1.06, *p*=.957)	
po2	Mean ± SD	110.7 ± 94.1	117.5 ± 101.7	po2	1.00 (1.00–1.00, *p*=.262)	
pco2	Mean ± SD	44.9 ± 13.8	44.5 ± 13.2	pco2	1.00 (0.99–1.01, *p*=.662)	
Total co2	Mean ± SD	25.7 ± 5.5	24.8 ± 5.4	totalco2	0.97 (0.94–0.99, *p*=.006)	0.99 (0.95–1.03, *p*=.668)

**Fig. 3:**
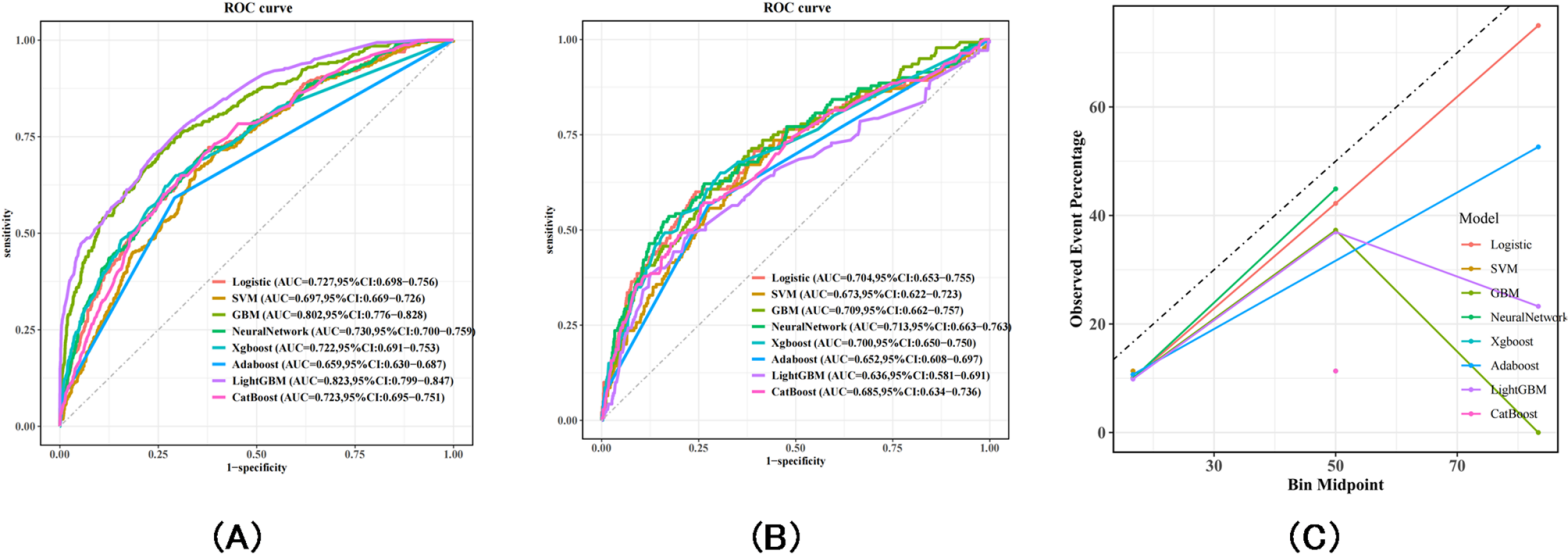
(**A**) ROC of imbalanced data training set. (**B**) ROC of imbalanced data internal validation set. (**C**) Calibration curves of imbalanced data internal validation set.

**Fig. 4:**
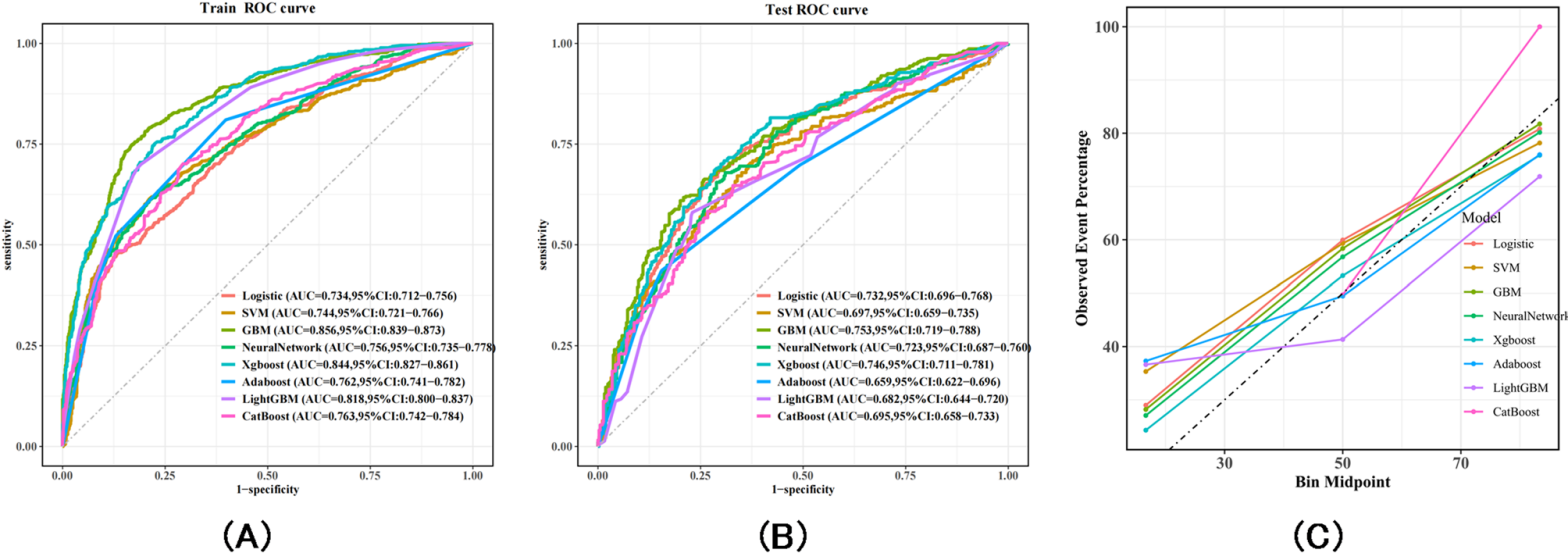
(**A**) ROC of the balanced data training set. (**B**) ROC of balanced data internal validation set. (**C**) Calibration curves of balanced data internal validation set.

**Fig. 5:**
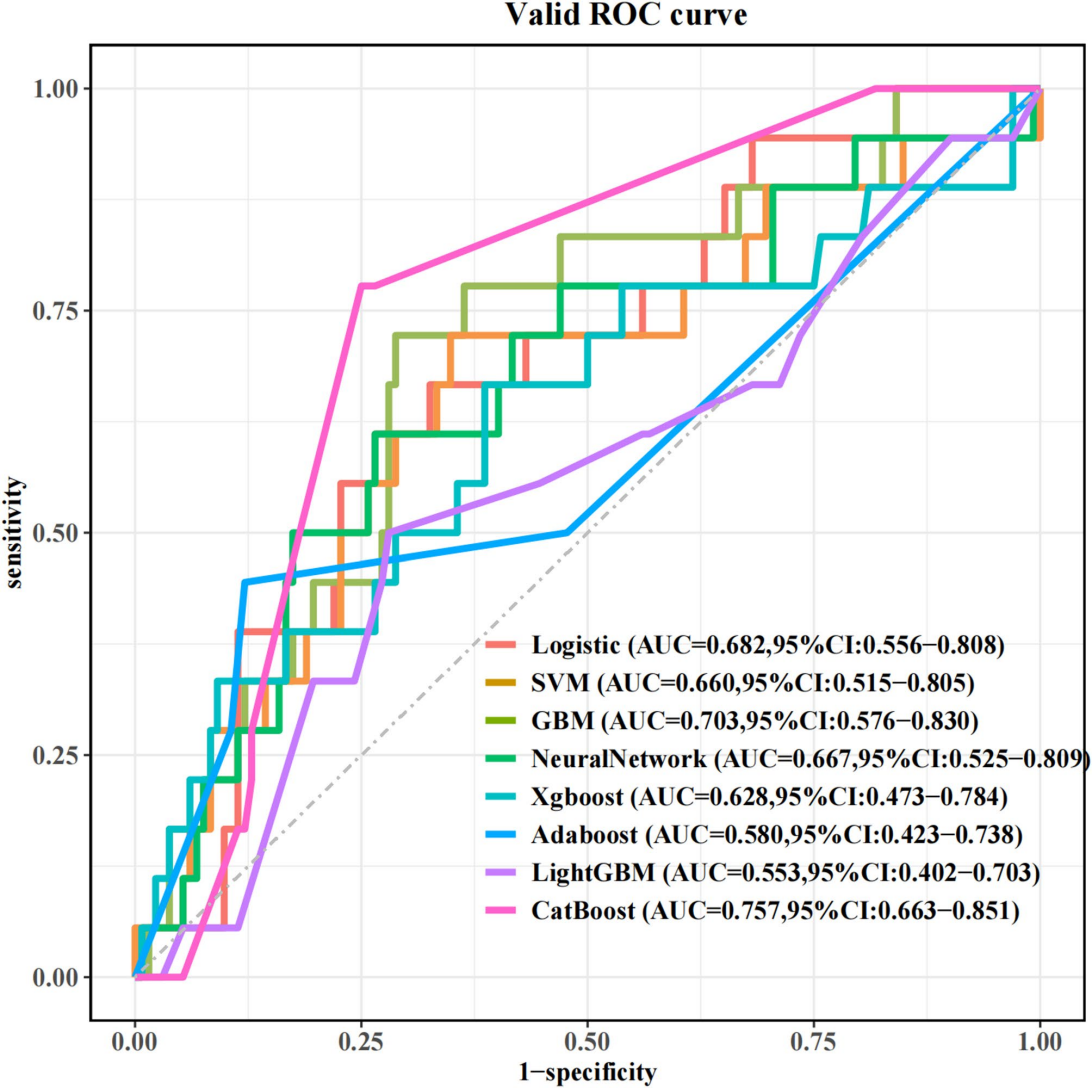
ROC of imbalanced data external validation set. This ROC curve shows the performance of different models on the external validation set with imbalanced data. Each curve represents the trade-off between sensitivity and specificity at various thresholds, with AUC values indicated for each model.

**Fig. 6:**
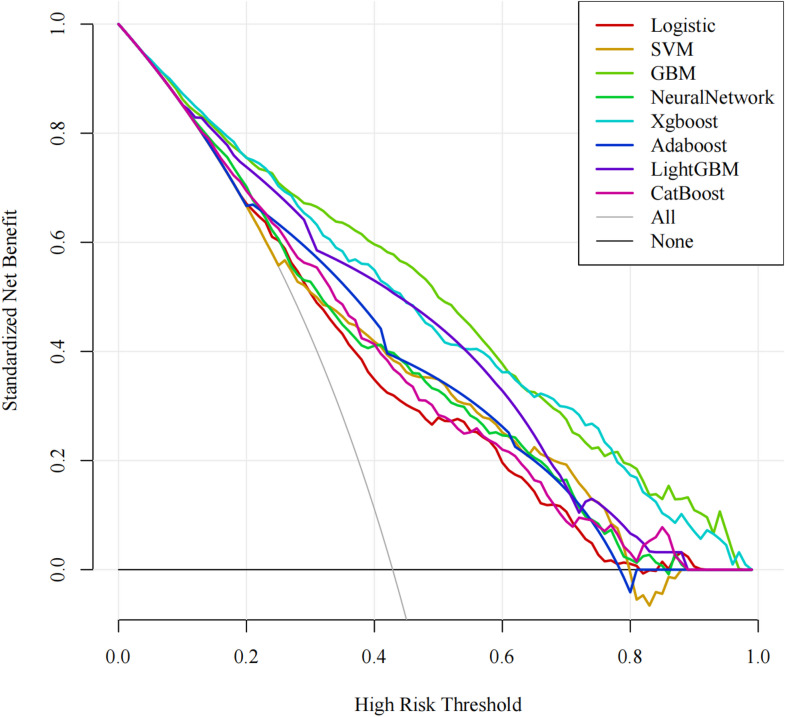
DCA curve of the training set.

**Fig. 7:**
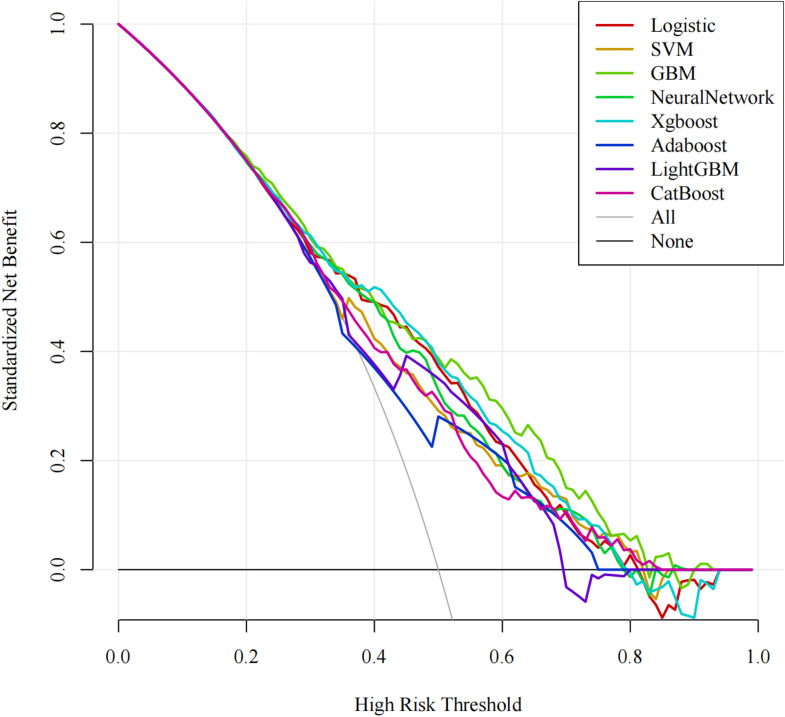
DCA curves of the test set.

**Fig. 8:**
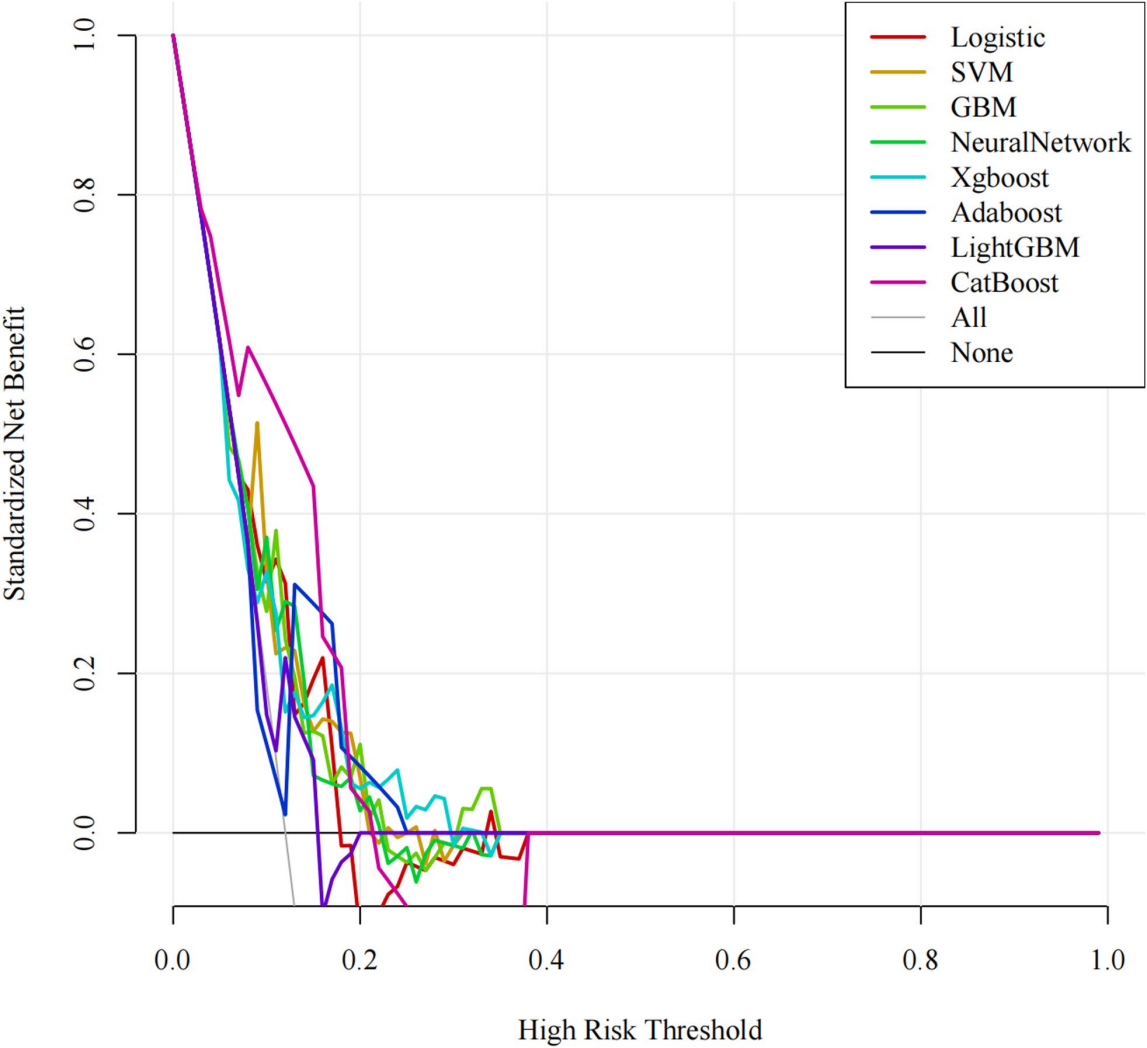
DCA curves of the external validation set. This figure presents the Decision Curve Analysis (DCA) for various models evaluated on the training set, testing set, and external validation set. The models include Logistic Regression, Support Vector Machine (SVM), Gradient Boosting Machine (GBM), Neural Network, XGBoost, Adaboost, LightGBM, and CatBoost. Each curve represents the change in standardized net benefit for each model at different high-risk thresholds, used to evaluate the model’s performance across various risk thresholds.

**Fig. 9:**
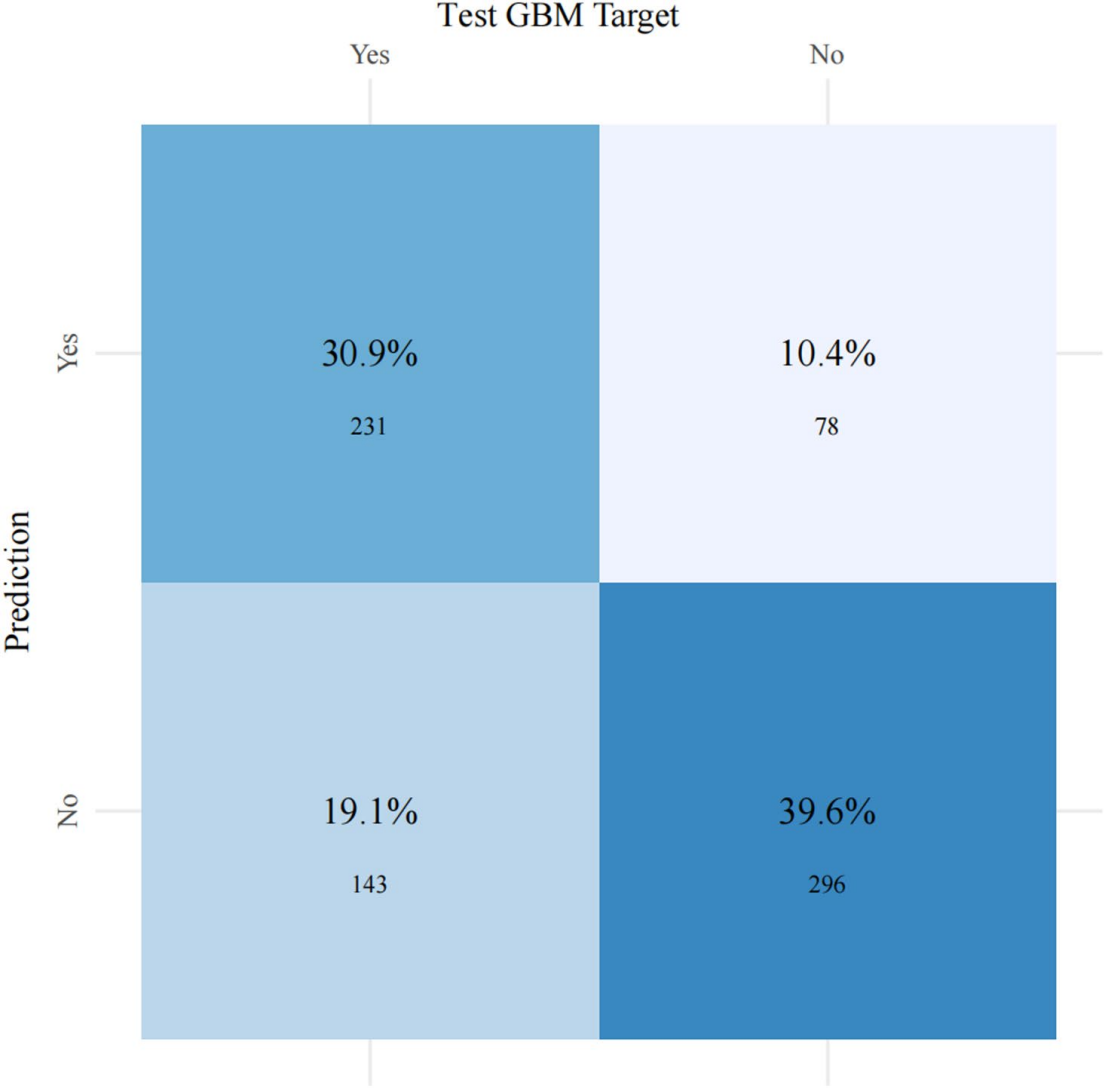
Confusion matrix for the internal validation set.

**Fig. 10:**
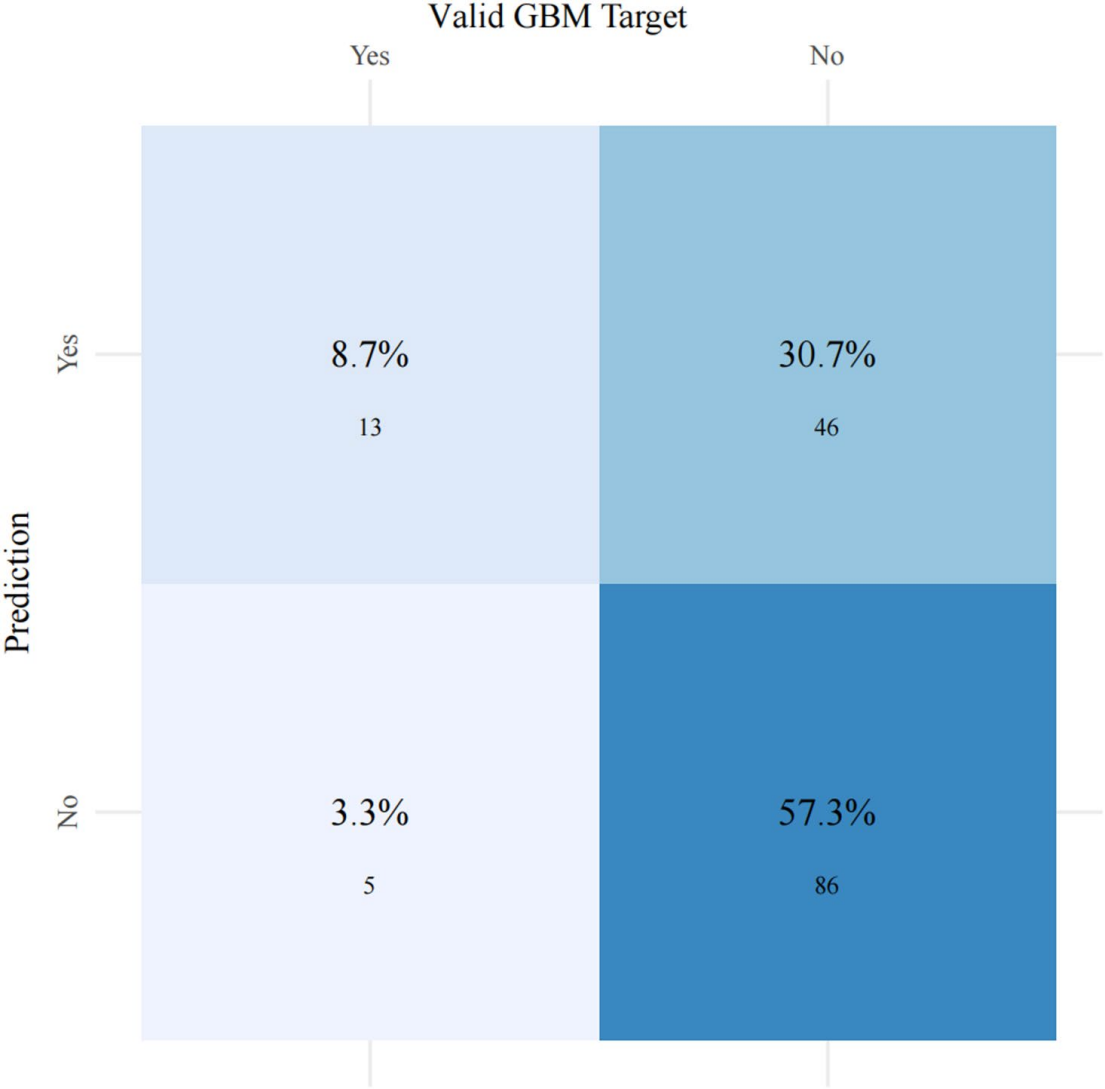
Confusion matrix for the external validation set.

In this study, we utilized eight machine learning models to predict the risk of bloodstream infections in ICU patients with pneumonia. To ensure the reliability of the model, we performed internal validation on the test set and external validation using patients from Dazhou Central Hospital. Overall, balanced models outperformed imbalanced models. On the imbalanced dataset, in the training set, LightGBM demonstrated the best performance (AUC = 0.823, 95% CI: 0.799–0.847), while the Neural Network model showed the best predictive performance in the internal validation set (AUC = 0.713, 95% CI: 0.663–0.763) **(**Fig. 3A, B**)**. After applying the SMOTE method for data balancing, the GBM and XGBoost models exhibited the strongest predictive performance, with training set AUCs of 0.856 (95% CI: 0.839–0.873) for GBM and 0.844 (95% CI: 0.827–0.861) for XGBoost, and internal validation set AUCs of 0.753 (95% CI: 0.719–0.788) for GBM and 0.746 (95% CI: 0.711–0.781) for XGBoost **(**Fig. 4A, B**)**. In external validation, the GBM model achieved an AUC of 0.703 (95% CI: 0.576–0.830) (Fig. 5).Calibration curves showed that, after SMOTE, the GBM model demonstrated reasonable agreement between predicted probabilities and observed event frequencies across the low-to-mid probability range, with slight deviation in the highest probability bins (Figs. 3C and 4C). Because the final predictor set included variables acquired after ICU admission, this model should be interpreted as a post-admission risk reassessment model. The Decision Curve Analysis (DCA) curve indicated that the model offers moderate clinical benefit **(**Figs. 6, 7 and 8**)**.Accuracy, precision, recall, and F1-Score metrics indicate that the model has moderate efficiency and effectiveness **(**Tables 4 and 5**)**.The confusion matrix visualizes metrics such as Accuracy, Precision, Recall, and F1-Score.**(**Figs. 9 and 10**)**.

**Table 4:** Test set accuracy, precision, recall, F1-Score and other index results.

Model	Threshold	Accuracy	Sensitivity	Specificity	Precision	F1
Logistic	0.381854758	0.697	0.693	0.701	0.698	0.695
SVM	0.293152526	0.666	0.709	0.623	0.653	0.679
GBM	0.429974096	0.705	0.618	0.791	0.748	0.676
NeuralNetwork	0.393522298	0.684	0.679	0.69	0.686	0.683
Xgboost	0.433927625	0.701	0.701	0.701	0.701	0.701
Adaboost	0.5	0.64	0.436	0.845	0.738	0.548
LightGBM	0.401889596	0.675	0.58	0.77	0.716	0.641
CatBoost	0.599301502	0.659	0.647	0.671	0.663	0.655

**Table 5:** Results of indicators such as accuracy, precision, recall and F1-Score of the external validation set.

Model	Threshold	Accuracy	Sensitivity	Specificity	Precision	F1
Logistic	0.385266454	0.72	0.611	0.735	0.239	0.344
SVM	0.342054909	0.66	0.722	0.652	0.22	0.338
GBM	0.322056677	0.713	0.722	0.712	0.255	0.377
NeuralNetwork	0.398991571	0.72	0.611	0.735	0.239	0.344
Xgboost	0.419680268	0.62	0.667	0.614	0.19	0.296
Adaboost	0.5	0.827	0.444	0.879	0.333	0.381
LightGBM	0.401889596	0.693	0.5	0.72	0.196	0.281
CatBoost	0.606112489	0.753	0.778	0.75	0.298	0.431

### Admission-only sensitivity analysis

**Fig. 11:**
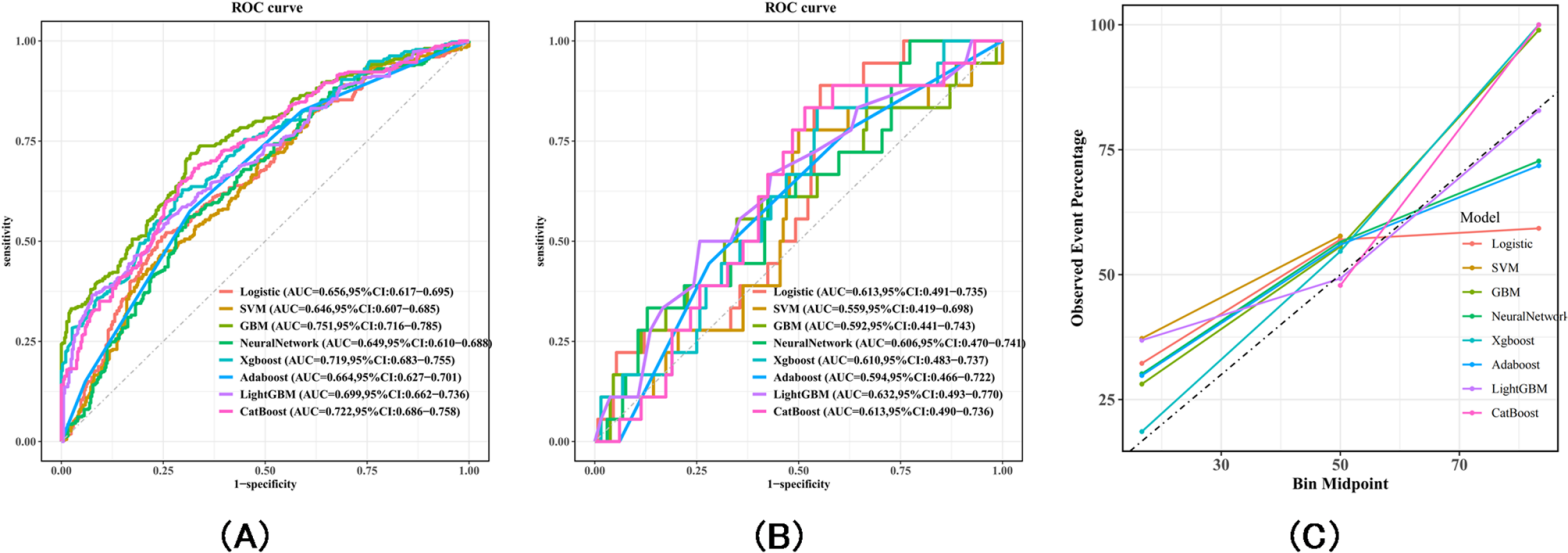
(**A**) ROC curves of the admission-only sensitivity analysis on the internal validation set. (**B**) ROC curves of the admission-only sensitivity analysis on the external validation set. (**C**) Calibration curves of the admission-only sensitivity analysis on the internal validation set.

To examine the extent to which the primary model depended on post-admission information, we performed an admission-only sensitivity analysis after excluding variables not available at or before ICU admission, including ICU length of stay and sputum culture results. Using the same model-development framework, the restricted model set showed attenuated discrimination compared with the primary analysis. The best-performing admission-only model achieved an AUC of 0.751 (95% CI 0.716–0.785) in the internal test set and 0.632 (95% CI 0.493–0.770) in the external validation set (Fig. 11A, B). Calibration analysis of the admission-only models showed acceptable agreement in the mid-range but overestimation at higher predicted probabilities (Fig. 11C).These findings indicate that model performance was reduced when only admission-available predictors were retained, underscoring the difficulty of early prediction without post-admission information.

### SHAP analysis and model interpretability

**Fig. 12:**
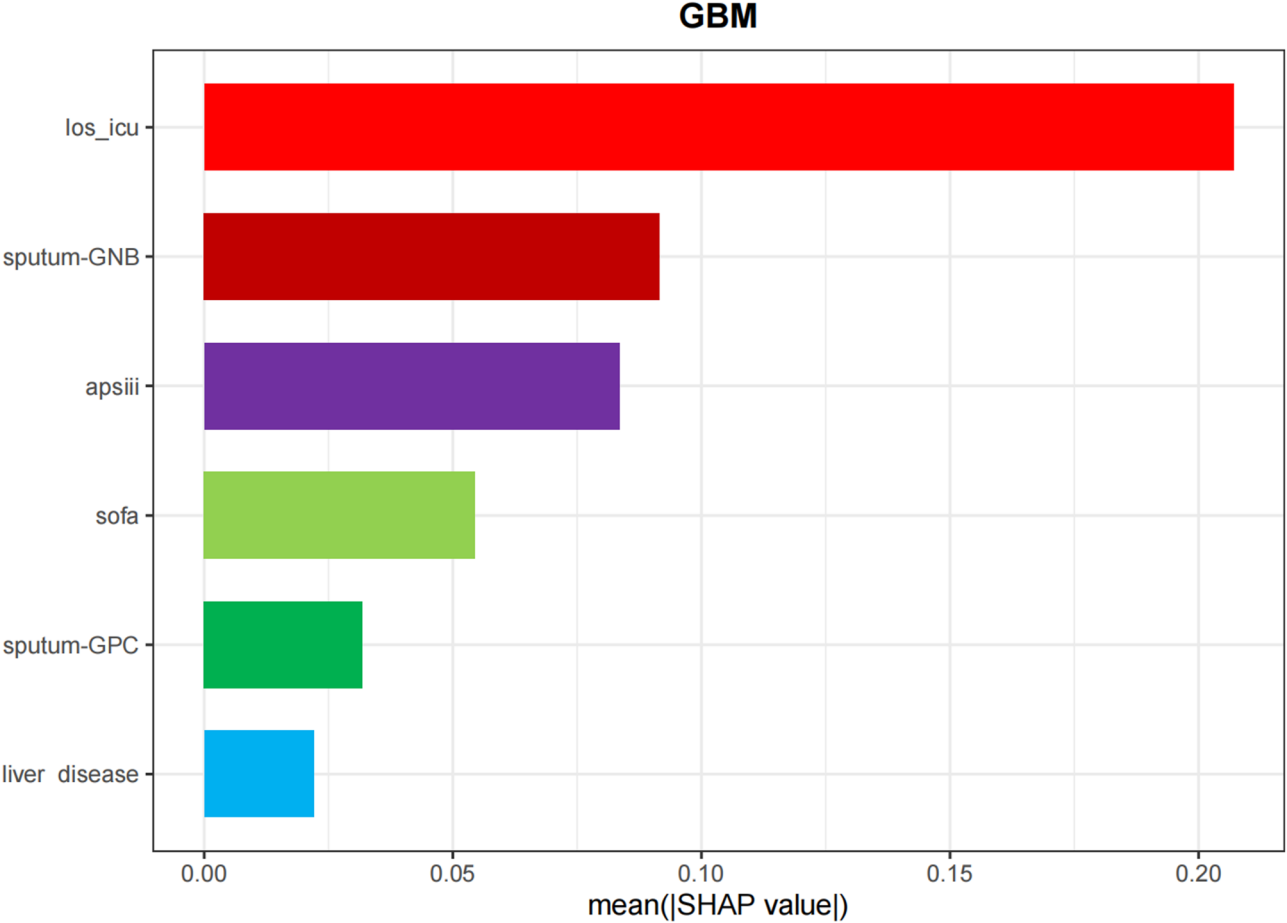
Feature importance of GBM model (based on SHAP values). This figure presents the average SHAP values of each feature in the GBM model, reflecting the contribution of different features to predicting bloodstream infection in ICU pneumonia patients. Features are ranked by importance, with higher SHAP values indicating that the feature has a greater impact on the model output.

To enhance the interpretability of the model, we used the SHAP algorithm to analyze the best model, the Gradient Boosting Machine (GBM) model. The SHAP histogram, shown in Fig. 12, reflects the contribution of each feature to the overall GBM model’s predictions. The larger the absolute SHAP value, the more important the feature is to the model output. Based on this ranking, ICU length of stay and sputum culture results were among the leading contributors to the fitted model’s predictions, alongside APSIII score, SOFA score, and liver injury. These findings should be interpreted as feature-attribution patterns within the post-admission model rather than direct evidence of causal or independently actionable effects.

**Fig. 13:**
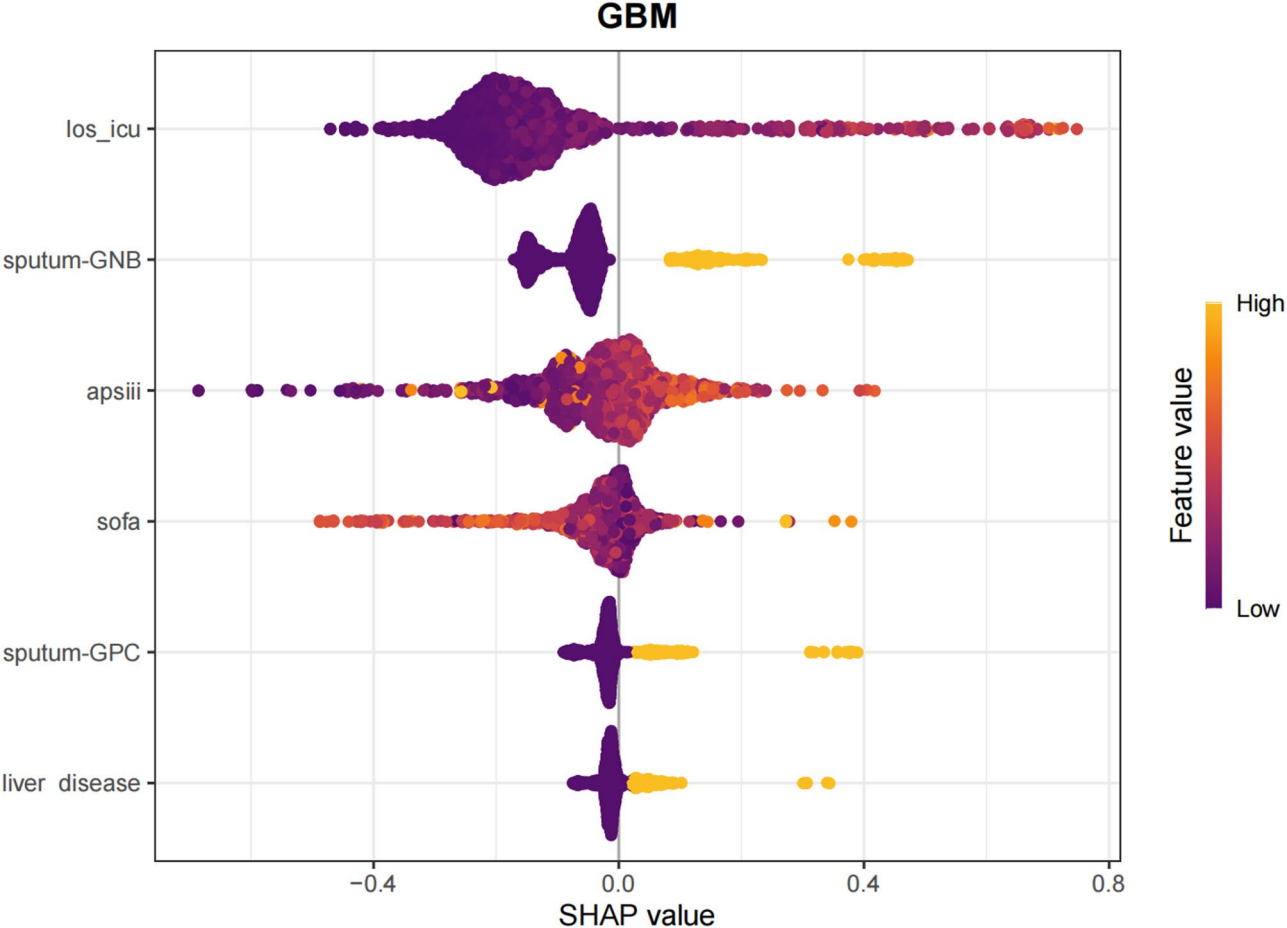
Distribution of SHAP values for GBM model features. This figure illustrates the distribution of SHAP values in the GBM model for key features used to predict bloodstream infection in ICU patients with pneumonia. Color indicates the high and low values of the features (purple for low values, yellow for high values).

Figure 13 displays the contribution of each clinical feature to the GBM model’s prediction, with the horizontal axis representing the SHAP values, indicating the direction and intensity of each feature’s positive or negative impact on the prediction. Each point in the figure represents a sample, and the color gradient from purple (low feature value) to yellow (high feature value) reflects the original value of the feature. The wider the distribution of points along the horizontal axis, the greater the influence of the feature on the model. The color-to-SHAP value correspondence reveals the trend of the feature value when driving the prediction result.

**Fig. 14:**
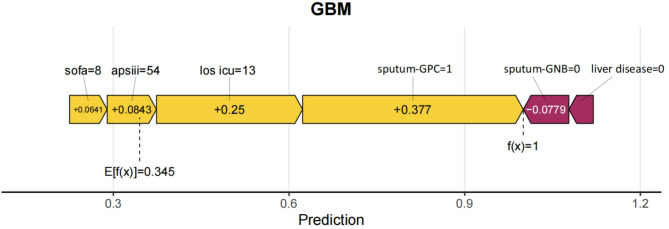
SHAP value interpretation of a single positive prediction in the GBM model.

Based on the SHAP value analysis, the GBM model revealed the impact of multiple key features on BSI prediction. For instance, in a patient who developed BSI, the SOFA score (SOFA = 8) contributed + 0.0843, Sputum-GPC positive (Sputum-GPC = 1) contributed + 0.377, and ICU length of stay (LOS_ICU = 13) contributed + 0.25, significantly driving the model’s prediction of BSI. As a result, the patient’s predicted risk value was 1, strongly indicating that they were a BSI-positive individual **(**Fig. 14**)**.

**Fig. 15:**
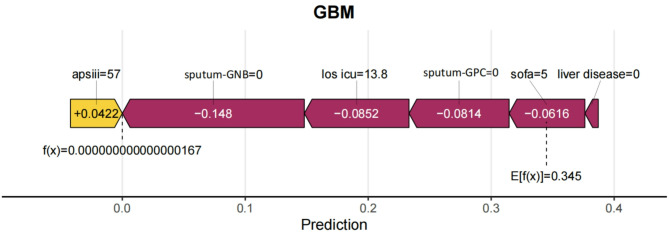
SHAP value interpretation of a single negative prediction in the GBM model.

In contrast, for a patient who did not develop BSI, the SOFA score (SOFA = 5) contributed − 0.0616, Sputum-GPC negative (Sputum-GPC = 0) contributed − 0.148, and APSIII score (APSIII = 57) contributed − 0.0852, significantly lowering the model’s predicted value. Ultimately, the predicted value for this patient was close to 0, strongly indicating they were a BSI-negative individual **(**Fig. 15**)**.

This figure shows the prediction results and SHAP value interpretation of GBM model for bloodstream infection in ICU patients with pneumonia. Through SHAP value, the contribution of various characteristics (such as SOFA score, APSIII score, length of ICU stay, sputum culture results, etc.) to the prediction of the model was clarified, and the patient was finally predicted to be a bloodstream infection.

This figure shows the prediction results and SHAP value interpretation of the GBM model for bloodstream infection in another ICU patient with pneumonia. Through SHAP value, the negative impact of various characteristics (such as APSIII score, length of ICU stay, sputum culture results, SOFA score, etc.) on the prediction was shown, and the final conclusion was that the probability of bloodstream infection in this patient was almost zero.

## Discussion

Bloodstream infection (BSI) is a severe healthcare-associated infection in pneumonia patients, often associated with high mortality rates and significantly increased hospitalization time and treatment costs^33–35^. To date, no externally validated model has been reported specifically for estimating BSI risk in ICU patients with pneumonia. In this study, we developed and externally validated an interpretable machine-learning model using variables available during the early ICU course to support post-admission risk reassessment. Among the evaluated algorithms, the Gradient Boosting Machine (GBM) model demonstrated the most favorable predictive value. By incorporating SHAP-based explanations, our model enables clinicians to understand which variables contribute most to BSI risk and how their effects vary across individual patients. However, given the moderate discrimination and limited external precision observed, the present model should be considered exploratory and hypothesis-generating rather than ready for direct clinical implementation.

In this retrospective study, we constructed the model based on the MIMIC database and validated it with the testing and external validation sets. Using easily accessible patient information and test indicators, we evaluated the risk of BSI in ICU pneumonia patients through eight machine learning algorithms. By comparing the AUC curves of the eight machine learning models, the Gradient Boosting Machine (GBM) model performed the best, with an AUC of 0.753 (95% CI: 0.719–0.788) in the testing set and an overall accuracy of 0.748. In external validation, the GBM model also showed good performance, with an AUC of 0.703 (95% CI: 0.576–0.830).

Furthermore, this study explored the role of SHAP-based algorithm interpretation in identifying risk factors for BSI prediction in ICU pneumonia patients. The analysis highlighted six key clinical features that significantly impacted the model’s output: ICU length of stay, sputum culture for Gram-negative bacteria (Sputum-GNB), APSIII score, SOFA score, sputum culture for Gram-positive cocci (Sputum-GPC), and liver injury.

In this study, ICU length of stay was identified as one of the key factors for predicting BSI in pneumonia patients. Existing literature has shown that ICU length of stay is closely associated with the occurrence of hospital-acquired infections^36,37^. Particularly in immunocompromised patients, extended hospital stays can increase the risk of infections^38^. Our findings also validate this, as longer ICU stays in pneumonia patients significantly increased the risk of BSI. However, ICU length of stay should not be interpreted as a simple baseline risk factor. As a time-dependent variable, it may partly capture time at risk, survivorship-related processes, and evolving clinical complexity during the ICU course. Therefore, an elevated model-predicted risk driven by prolonged ICU stay may support closer monitoring, rather than serving as direct evidence of an independent causal effect.

In addition, the APSIII score, as a tool to assess the severity of illness and prognosis in ICU patients, also demonstrated its significance in this study^39^. The APSIII score is a comprehensive assessment of a patient’s physiological indicators within the first 24 h of ICU admission and effectively reflects the patient’s clinical status^40,41^. Previous research has shown that patients with higher APSIII scores are more likely to develop complications, including BSI (Wang et al., 2020). Our results further support this, with higher APSIII scores indicating a significantly increased risk of BSI. Studies have also shown that higher SOFA scores are linked to an increased likelihood of severe complications, including BSI^42–44^. Our study corroborates this conclusion, finding that higher SOFA scores significantly raised the risk of BSI, suggesting that SOFA scores can serve not only as a clinical disease severity measure but also as a key indicator for predicting infection risk in ICU patients.

Sputum culture positivity, as another important predictor, was closely associated with the occurrence of BSI. A positive sputum culture typically indicates the presence of bacterial infection, with the type of bacteria potentially influencing the severity and transmission route of the infection^45^. In this study, patients with positive sputum cultures were more likely to develop BSI, consistent with previous research, indicating that these patients should be particularly cautious about the risk of bloodstream infections^20,46,47^.

Liver disease not only worsens a patient’s overall condition but also suppresses immune function, thereby increasing susceptibility to infections^48,49^. Our study found that patients with liver disease had a higher risk of BSI, highlighting the importance of early identification and intervention for these patients.

Globally, approximately 70% of ICU patients use antibiotics, and during the first 24 h after the occurrence of BSI, the proportion of patients receiving inappropriate or no antibiotic treatment is as high as 47.3%^50,51^. Excessive antibiotic use within ICUs is also a significant issue.

Previous studies have attempted to predict the occurrence of BSI. In 2004, Mark L. published a study on predicting pneumonia bacteremia, identifying independent risk factors for pneumonia-related bacteremia, including antibiotic treatment, liver disease, abnormal temperature, white blood cell count, heart rate, blood pressure, and urea nitrogen^52^. Ratzinger et al. proposed a machine learning (ML)-based model for predicting BSI in non-ICU hospitalized patients, incorporating several standard laboratory indicators, including procalcitonin, C-reactive protein (CRP), and 13 pro-inflammatory and anti-inflammatory cytokines. Using the random forest (RF) model, they achieved the best prediction results, with an AUROC value of 0.74 (95% CI 0.61–0.87)^53^. Additionally, a meta-analysis in 2024 reviewed the major risk factors for pneumonia-related bacteremia, with substantial variability across studies, including laboratory indicators and clinical manifestations. Major risk factors included CRP > 17 mg/dL, albumin < 3.3 mg/dL, platelet count < 130,000/µL, sodium < 130 mmol/L, and blood urea nitrogen (BUN) > 30 mmol/L^54^. However, these studies focus on predicting BSI in all ICU patients or community-acquired pneumonia bacteremia. Our study, for the first time, targets the specific subgroup of ICU pneumonia patients, incorporating sputum culture results and ICU length of stay as predictive factors to assess the risk of BSI occurrence during their ICU stay. Especially when ICU pneumonia patients have prolonged ICU stays and experience false-negative blood culture results, our predictive model can enhance clinicians’ confidence in decision-making.

Despite the promising findings, several methodological considerations must be acknowledged. The present study used a static prediction framework and did not explicitly model time-varying exposures, competing events, or subgroup heterogeneity. Because ICU patients with pneumonia may follow heterogeneous clinical trajectories, future work should evaluate dynamic prediction strategies, landmark-based designs, and, where feasible, subgroup-specific models to better capture the evolving nature of BSI risk^55^.

Furthermore, it is crucial to emphasize that this study was predictive rather than causal. Accordingly, the model output should not be interpreted as direct evidence for blood-culture decisions or antibiotic escalation. Any intervention strategy guided by model-predicted risk would require dedicated causal-evaluation frameworks, such as target trial emulation, in future research to determine whether model-guided strategies actually improve patient outcomes^56^.

Based on six clinical features, including ICU length of stay, sputum culture results, APSIII score, SOFA score, and liver injury, we developed the GBM model to predict the risk of BSI. The model performed well in both the testing and external validation sets, with AUCs of 0.753 and 0.703, respectively.The features included in our model are easily accessible in clinical practice, offering a novel approach for post-admission risk reassessment. However, as noted above, the model’s current precision limits its direct clinical utility. Substantial refinement and further prospective validation are required before this predictive framework can be safely integrated into routine clinical decision-making.

## Conclusion

In conclusion, we developed and externally validated an interpretable machine-learning model for post-admission reassessment of BSI risk in ICU patients with pneumonia. Although the primary model showed moderate discrimination, its limited external precision and the weaker performance of the admission-only sensitivity analysis indicate that substantial refinement and further validation are required before clinical implementation.

## Limitations

Several limitations should be noted regarding the present study. First, the model was developed and externally validated using data from a single hospital, which may limit its generalizability across different institutions. Second, due to the high proportion of missing values in the MIMIC-IV database, several clinically relevant variables (e.g., complete blood count, blood gas analysis, C-reactive protein, and procalcitonin) were excluded. Furthermore, while we employed the robust Multiple Imputation by Chained Equations (MICE) method, this approach relies on the assumption of Missing At Random (MAR), and a formal sensitivity analysis under alternative imputation strategies was not conducted. Third, the use of SMOTE for oversampling may increase the risk of model overfitting, highlighting the need for further validation with larger, real-world clinical datasets. Fourth, we did not distinguish between different types of pneumonia (e.g., community-acquired versus hospital-acquired), which may have introduced unmeasured confounding. Fifth, regarding the model’s clinical applicability, the primary model incorporated post-admission variables (including ICU length of stay and sputum culture results), which limits its use as an admission-time decision tool. Some retained predictors were time-dependent and may reflect evolving clinical processes rather than baseline causal effects. Moreover, the study used a static prediction framework and did not explicitly model time-varying exposures, competing events, or subgroup heterogeneity. Because ICU patients with pneumonia may follow heterogeneous clinical trajectories, future work should evaluate dynamic prediction strategies, landmark-based designs, and subgroup-specific models. Finally, the study was predictive and associational rather than causal; therefore, whether model-guided blood-culture or antibiotic strategies improve patient outcomes remains unknown and would require dedicated causal-evaluation designs, such as target trial emulation, in future prospective multicenter studies.

## Data Availability

The datasets used and/or analysed during the current study available from the corresponding author on reasonable request.

## Abbreviations

BSI: Bloodstream Infection
ICU: Intensive Care Unit
AUC: Area Under the Curve
Sputum-GNB: Sputum Culture for Gram-Negative Bacteria
Sputum-GPC: Sputum Culture for Gram-Positive Cocci
SHAP: Shapley Additive Explanations
